# Health visiting teams and children’s oral health: a scoping review

**DOI:** 10.1186/s12903-022-02611-6

**Published:** 2022-12-10

**Authors:** Sarab El-Yousfi, Zoe Marshman, Patricia N. Albers, Samantha Watt, Ruth Kipping, Joanna G. Williams

**Affiliations:** 1 School of Clinical Dentistry, University of Sheffield, Sheffield, UK; 2 Population Health Sciences, Bristol Medical School, University of Bristol, Bristol, UK

**Keywords:** Health visitors, Health visiting teams, Children, Oral health, Review

## Abstract

**Background:**

Recent policies have recommended early-life interventions to prevent caries. The four nations of the UK each have a national universal children’s health programme, through which health visitors and their wider team (HVTs) promote health in the early years. HVT visits offer an opportunity to support parents to improve their child’s oral health. A scoping review was conducted to provide a descriptive synthesis of the current literature related to the role of HVTs in improving the oral health of children 0–5 years old and to identify significant gaps for future research. This review informed the feasibility study of the First Dental Steps Intervention, a targeted health visitor-led infant oral health intervention in England.

**Methods:**

Electronic database searches for peer-reviewed literature were performed using Medline via Ovid and Web of Science (1946–2021). The quality of included intervention studies was assessed using the Effective Public Health Practice Project tool. Additionally, a grey literature search was conducted (key organisations, bibliographic and thesis databases, forwards and backwards citation, Google).

**Results:**

Thirty-nine publications, published between 1980 and 2021, were included. The majority of included papers were from the UK. The quality of intervention studies (n = 7) ranged from weak to strong. Thematic analysis identified the following themes: (1) professional knowledge, education, and training; (2) involvement of HVs in the delivery of oral health interventions; (3) effectiveness of interventions; (4) perspectives of HVs providing oral health advice and acceptability; and (5) barriers and facilitators to promoting oral health. The grey literature search identified 125 sources. HVT involvement was reported in a variety of source types: reports, guidance documents, evaluations, reviews, and training resources. HVTs were involved in oral health by providing oral health packs, brushing and oral health advice, registration and attendance, oral health training, risk assessment, and referral to dental services.

**Conclusion:**

The current literature suggests that HVTs are well placed to improve children’s oral health. Facilitators and barriers are encountered by HVTs in promoting oral health which should be considered by commissioners. There is a need for future high-quality studies that address the inadequacies found and provide further evidence of the effectiveness of HVT’s oral health interventions.

**Supplementary Information:**

The online version contains supplementary material available at 10.1186/s12903-022-02611-6.

## Background

Dental caries is the most prevalent preventable disease in children worldwide [1]. Caries can have a significant impact on the daily lives of children and their families. Children may suffer from pain potentially hindering their ability to eat or sleep [2, 3]. Furthermore, caries may have a detrimental effect on a child’s speech development [4], general health [5, 6], educational attainment through missing school days [7], and overall quality of life [8]. Consequently, dental caries poses a significant burden on children, families, wider society, and health services. In the UK, providing dental care under general anaesthetic is the primary reason for hospital admission of young children [9]. Therefore, primary prevention of dental caries in children is a major priority for public health commissioners [10]. As a result of the Health and Social Care Act, 2012 local authorities (LAs) have a statutory responsibility for commissioning oral health improvement programmes. The four nations of the UK each have a national universal children’s health programme, through these programmes HVTs support health improvement in the early years and are expected to promote oral health (Additional file 1: Appendix 1). For example, in England the national Healthy Child Programme (HCP) focuses on improving the health of children and young people aged 0–19 years. [11]. This is an early intervention and prevention programme aimed at supporting ante-natal mothers and children during the early years of child development. The 0 to 5-year element of the HCP is primarily led by health visitors (HVs) and their wider teams. Health visitors and their wider teams are often referred to as health visiting teams (HVTs). Recent policies have recommended early-life interventions to prevent caries [9, 12] and HVT visits offer an opportunity through which parents may be supported to improve their child’s oral health. As part of the HCP, the HVTs carry out five universal mandated developmental checks. HVTs are required to discuss good oral health practices and provide oral health advice during the fourth developmental check (child is aged 12 months), along with other topics they normally discuss such as general child development, diet, obesity prevention, and safety [13, 14]. The latest guidance now recommends oral health advice be given at all of the five mandated developmental checks (including newborn, 6–8 weeks, and 2–2.5 year check).

Despite this, there has been little exploration of the role of HVTs in improving children’s oral health. Therefore, a scoping review was conducted to synthesise and analyse the existing peer-reviewed and grey literature on HVTs and children’s oral health. This review was part of a wider study and was undertaken primarily to inform the evaluation of an oral health intervention. The aim of this scoping review was to describe the available literature related to the role of HVTs in improving the oral health of children 0–5 years old and to identify significant gaps in the current literature which would benefit from further research.

## Methods

Scoping reviews are a method of reviewing the literature to create a broad overview of a research topic, mapping out key themes, and identifying research gaps. They serve to present a narrative account of current literature and map the evidence available [15]. Scoping reviews are used to inform practice, policy, and research [16]. Although they differ from systematic reviews in that they do not provide detailed answers to a specific research question they must still be conducted rigorously and transparently to ensure trustworthiness [17].

This scoping review was conducted using the Arksey and O’Malley five-stage framework [15] with Levac et al. enhancements [18] which provide specific recommendations for each stage of the framework [15] to improve methodological rigour. The results of this scoping review have been reported according to the Preferred Reporting Items for Systematic reviews and Meta-Analyses extension for Scoping Reviews [19] (Additional file 2: Appendix 2).

### Stage 1. Identifying the research question

To generate a breadth of coverage, the following research questions were formulated for the review: ‘What is known about the contribution of HVTs in improving the oral health of children aged 0–5 years?’ and ‘What is known about oral health interventions for children aged 0–5 years that involve HVTs?’.

### Stage 2. Identifying relevant studies

The searches for the peer-reviewed and grey literature were conducted independently. The grey literature search was limited to the UK whereas the peer-reviewed literature search was not.

#### Peer-reviewed literature

Relevant studies were searched for in peer-reviewed publications using electronic databases including Medline via the Ovid interface and Web of Science within a date range of 1946–2021. A detailed search strategy was designed using keywords to retrieve relevant literature. The search strategy was developed for Medline via the Ovid interface (Additional file 3: Appendix 3) and was revised for the other platform. Search terms were searched for in the title and/or abstract and topic heading as appropriate. All searches were conducted in June 2021. A pilot search was conducted by one reviewer (SE) to assess the appropriateness of the search terms in generating relevant results. The search strategy was discussed with the research team and was deemed appropriate, and the number of included papers was found to be manageable within the timescales of the study. The output references of both searches were exported into Endnote X9. Duplicates were then recorded and removed. Furthermore, the reference lists of all included studies were searched for relevant studies.

#### Grey literature

For this scoping review, grey literature was defined as “that which is produced on all levels of government, academics, business and industry in print and electronic formats, but which is not controlled by commercial publishers” [19]. Examples of grey literature included: websites, theses, reports, conference proceedings, and policy documents [20].

The following search strategy was used to find sources of grey literature regarding HVT involvement in the oral health of children:

1. Eighteen key organisations suggested by experts and academics working in public health (see Additional file 3: Appendix 4).
2. A broad, iterative search of seventeen bibliographic and thesis databases suggested by an Information Specialist (see Additional file 3: Appendix 5).
3. Forwards and backward citation searching of articles included in the peer-reviewed, published literature scoping review.
4. A search using the internet search engine Google. Five searches were conducted using the advanced search function in incognito mode; the search was limited to sources in the English language and on HVTs involvement in oral health in the UK (see Additional file 3: Appendix 6).

A pragmatic decision was taken to limit the grey literature search to the UK based on almost all of the resulting citations from the peer-reviewed literature search being from the UK. All the searches were conducted between July and August 2021.

### Stage 3 Study and information selection

#### Peer-reviewed literature

The review process consisted of two levels of screening. Initially, one reviewer (SE) screened the title and abstract of all retrieved citations against the inclusion/exclusion criteria shown in Table 1. All potentially relevant studies were retrieved for full-text assessment. These were then included in a full-text review by two independent reviewers (SE, PA) to assess if they met the inclusion/exclusion criteria. Any disagreement on eligibility was resolved through discussion including a third reviewer (ZM) and resolved by consensus.

**Table 1:** Inclusion and exclusion criteria

*Inclusion criteria*	
Peer-reviewed publications (all research designs)	
Focus is related to the involvement of HVTs in improving the oral health of children aged 0–5	
*Exclusion criteria*	
Articles unavailable in English	
Articles unavailable in full text	
Articles such as editorials, commentaries, and opinion pieces	

Inclusion and exclusion criteria were decided a priori by the research team and were applied to the studies. All publications irrespective of study design in which the focus related to the involvement of HVTs in improving the oral health of children aged 0–5 were included. There are various terminology and different structures in place globally for health visiting, thus the primary focus was the UK literature. However, the review was not limited to the UK to increase the sensitivity of the search. Studies that were not available in English or in full text were excluded. Additionally, publications such as editorials and commentaries were excluded; however, their reference lists were searched for the original studies.

#### Grey literature

One researcher (SW) screened sources by title and contents page and determined the potential relevance of the sources. A second researcher (PA) duplicated searches of three of the eighteen key organisations and one of the five Google searches to ensure the validity and replicability of the search. PA then shared files of the results of the searches. These files were checked by SW and no additional potentially relevant sources were identified.

All searches conducted were saved in an Excel file with the search date, search terms used, limitations applied, and the number of results documented. The title of all potentially relevant sources and the corresponding Uniform Resource Locator were also stored in Excel. Following completion of the searches, all sources deemed potentially relevant were saved to an EndNote library to be read in full and eligibility assessed. Deduplication was conducted using EndNote.

Inclusion and exclusion criteria were decided a priori by the research team and were applied to the sources. Sources were included if they were freely and publicly available and were about HVT’s involvement in improving the oral health of children aged 0–5 in the UK. Sources were excluded if they were not in English, an opinion piece, personal blog, reflection post, media report, or meeting agenda. There were no date restrictions placed.

### Stage 4 Data charting

#### Peer-reviewed literature

The data from eligible full-text articles were extracted, charted, and summarised by the reviewers using a bespoke Microsoft Excel spreadsheet using the variables shown in Table 2. The data extraction form was piloted independently by reviewing three articles by each reviewer (SE, PA). This was then discussed with the wider research team (ZM, RK, JW) to ensure the data extraction form was appropriate in capturing the outcomes of interest and the data extraction form was refined accordingly. A further two articles were then reviewed independently (SE, PA), and the extraction spreadsheet was deemed suitable. Each reviewer (SE, PA) then independently reviewed half of the included studies which were then validated by the other reviewer.

**Table 2:** Variables captured for peer-reviewed literature

Details of the publication: author(s), title, year of publication, journal, country affiliated to the lead author	
Details of the study: type of study design, study aim, sample characteristics (target group, total number, age range), setting	
Details of the intervention (where applicable): Intervention aim & components, main outcome measure, key findings and recommendations	

#### Grey literature

Once searches were complete, the researchers conducted a meeting and discussed the nature of the sources. As the search yielded a variety of source types it was decided that two separate data charting forms were needed to chart and map different information depending on the source type. The research team developed two data charting forms and discussed which variables to chart from the sources and appropriate headings shown in Table 3. One data charting form was made for reports, policy documents, guidance documents, training resources, and reviews. A separate data charting form was prepared for case studies and evaluations, this form included study-specific details such as the study design, aims, and outcome measures employed. One researcher (SW) completed all of the data charting.

**Table 3:** Variables captured for grey literature

Details of the source: author(s), title, year of publication, document type, name of programme	
Details of the programme: name, country, regional or national, launch year and duration, universal or targeted, HVTs involvement	
Details of the intervention: nature of intervention, intervention details, cost of intervention(s) reported (Y/N), cost-effectiveness reported (Y/N), effectiveness reported (Y/N), aims and objectives, outcome measures, impact on health inequalities discussed (Y/N), target audience of resource	

### Stage 5 Collating, summarising, and reporting results

#### Peer-reviewed literature

The data were extracted and collated, with quantitative and qualitative studies synthesised separately. Quantitative data were presented descriptively, and qualitative data presented as themes. For this stage, a thematic mapping approach [17] was undertaken to summarise the key findings enabling a narrative account of the existing literature concerning the key areas of the role of HVTs and children’s oral health. A similar method was used for the grey literature.

#### Quality assessment of peer-reviewed literature

A quality assessment of the peer-reviewed intervention studies was conducted using the Effective Public Health Practice Project tool (EPHPP). This tool may be used to evaluate a variety of intervention study designs and has been reported to have content and construct validity [21, 22]. The tool evaluates six domains: selection bias, study design, confounders, blinding, data collection method, and withdrawals/dropouts. Each domain was rated and given a score ranging from strong (3), moderate (2), or weak (1). The average of all domain scores was calculated to give the study a total quality score. Studies were then assigned a quality rating of strong (2.51–3.00) moderate (1.51–2.50) or weak (1.00–1.50) according to their total score. This was conducted by two independent reviewers (SE, PA) and any discrepancies in quality ratings were resolved by discussion. As mentioned previously this review was conducted essentially to inform the evaluation of an intervention therefore this review focused only on assessing the quality of the intervention studies.

## Results

### Results of peer-reviewed literature

#### Characteristics of the included studies

**Fig. 1:**
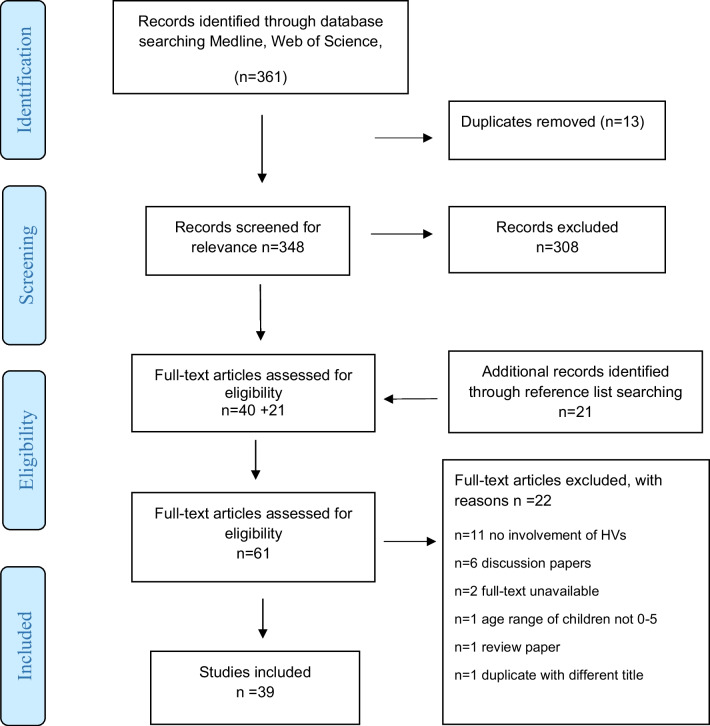
PRISMA-ScR flowchart for the peer-reviewed literature

Overall, 39 articles were included in the final analysis (Additional file 1: Appendix 7) with the process for study inclusion described in the he Preferred Reporting Items for Systematic Reviews and Meta-Analyses-Extension for Scoping Reviews (PRISMA_ScR) flow chart (Fig. 1).

Publication dates of the included studies ranged from 1980 to 2021 with an increase in the number of publications over the past decade with 44% published between 2010 and 2021. The remaining 56% were published over three decades; with 15% between 2000 and 2009, 26% between 1990 and 1999, and 15% between 1980 and 1989. The majority of included papers were from the UK with one article from Australia and one article from the USA. Some publications were regarding the same study with four publications regarding the Childsmile national oral health improvement programme (OHIP) [23–26] and three regarding the NAP SACC UK study [27–29]. In terms of methodological design, most papers were cross-sectional (41%, n = 16) [30–45]. Among the remaining papers there were four randomised controlled trials (RCTs) [28, 46–48], three RCT protocols [27, 49, 50], one process evaluation [29], one mixed- method study [51], four qualitative studies [52–55], two evaluation papers [23, 25], two feasibility studies [56, 57], one before and after study [58], one quasi-experimental study [59], and one economic evaluation [26].

The quality assessment included seven intervention studies (Additional file 1: Appendix 8). Based on the assessment of the selected studies, two studies had a quality rating of strong, three studies were rated as moderate, and two studies were rated as weak. The lowest scoring domain was found to be selection bias (which included both identification of study participants and consent to participate).

#### Synthesis of study findings

Very few of the included studies used the term health visiting teams with the majority using the term health visitors. Thematic analysis of the papers identified the following themes: (1) Professional knowledge, education, and training; (2) Involvement of HVs in the delivery of oral health interventions; (3) Effectiveness of interventions; (4) Perspectives of HVs providing oral health advice and acceptability; and (5) Barriers and facilitators to promoting oral health. There were many cross-cutting themes in the included articles.

##### Professional knowledge, education, and training

Knowledge, previous education, and training of HVs in oral health was a central theme of the included studies with many studies recognising the need for HVs to access ongoing, formal training, post-graduation, to develop the skills and confidence enabling them to promote oral health [31, 32, 36–38, 51, 52, 54, 55, 58].

Overall, the data from the studies indicated that HVTs had a good general knowledge of oral health [32, 36, 41, 51, 52, 55]. Nonetheless, several gaps in knowledge were identified particularly regarding fluoride guidelines, such as toothpaste concentrations and amounts [32, 36, 41, 51, 55, 58], dental issues during pregnancy [37], various aspects regarding toothbrushing such as toothbrushing techniques [37], the time interval between evening meal/feeding and brushing [55], advice on behaviour management to facilitate brushing in very young children [55] and the caregivers’ role in assisting and supervising children’s tooth brushing [58].

Only two studies directly investigated the education provided for HVs with the most recent publication from more than two decades ago [38, 42]. It was reported that most courses in health visiting did include some form of dental health training; however, in more than one-third of the educational institutions this was provided by non-dental professionals [38]. Sixteen years prior to this, it was indicated that dental health training varied considerably from one educational institution to another regarding content, syllabus, and a lack of standard requirements [42].

##### Involvement of HVs in the delivery of oral health interventions

Several oral health interventions for children aged 0–5 years involved HVs. The role of HVs involved providing oral health advice [23–25, 31, 36, 41, 47, 48, 50, 59–61], providing families with toothbrushing packs and other resources [23–25, 30, 47, 48, 50], promoting dental registration and access to dental services [23–25, 59, 60] and supporting nurseries to review policies and practices and work towards improving nutrition and oral health of children [27–29].

A study investigating a pilot dental outreach service for hard to reach families made use of home visits and collaboration with a familiar community nurse [57]. The study found that the inclusion of the community nurse helped alleviate the fears and uncertainties the families had about attending dental treatment.

One study included in this review did not directly involve HVs in the delivery of the intervention [46]. Their role involved assisting with the recruitment of preschool children in a clinical trial investigating the effectiveness of glass ionomer sealants. At mother-baby clinics, HVs provided parents with information regarding the trial and referred them to the research team for a screening appointment.

##### Effectiveness of intervention

Effectiveness was explored in the included studies according to different outcome measures such as knowledge gained by HVs and/or parents as a result of the intervention, increased levels of dental registration, levels of recall of received oral health advice, and reduction in dental caries.

Providing HVs with an educational intervention that included oral health knowledge and motivational interviewing components was reported to have successfully improved both oral health knowledge and communication techniques with families [58].

The adoption of a community-based approach was reported to facilitate dental registration and access to dental services for preschool children living in areas of high deprivation [59]. However, another study found that there was no significant improvement in dental registrations and dental attendance [60].

One study investigated the effectiveness of an oral health intervention in terms of recall of advice provided. They reported a significant improvement in mothers’ recall of the advice, given by health visitors, regarding the use of a feeder cup instead of a bottle, brushing their babies’ teeth with fluoride toothpaste, restricting sugary foods and drinks, the use of sugar‐free medicine and registering babies with a dentist [30].

An RCT examined the effect on dental caries of a specially trained HV in dental health delivering oral health education [47]. There was no statistically significant difference in mean dmfs scores at five years between the two groups. The authors suggest that examining the children in the control group at three years may have led to cross-contamination by focusing attention on oral health care. Consequently, rendering it less likely to detect a significant difference between the two groups.

Another RCT investigated the effectiveness of a multi-stage oral health promotion programme in reducing early childhood caries (ECC) on a community level [48] and reported the intervention was successful in reducing early childhood caries in children in the test group as compared to the control group. However, on a community level, there was no significant difference found in the prevalence of ECC between the two groups. The authors attribute this to the high proportion of children who did not participate in the programme. The findings of the trial also indicate that parents were more likely to report the adoption of three positive oral health behaviours: the cessation of bottle use, use of sugar-free drinks, and brushing twice daily.

##### Perspectives of health visitors providing oral health advice and acceptability

A small group of studies explored the views and experiences of HVs surrounding the delivery of oral health promotion this included survey studies and qualitative studies providing more in-depth data.

There was general agreement that HVs viewed oral health as important and a survey conducted by Oge et al. [32] reported that almost all HVs (99.8%) believed that promoting oral health should be incorporated into their developmental checks. Furthermore, the HV respondents viewed oral health as part of general health (99.5%) and tooth decay in primary teeth as important (97%). Another study reported that a few HVs expressed concern that they were already overworked and that promoting oral health would add to their list of responsibilities [30].

Toothbrushing packs (containing toothbrush, fluoride toothpaste, and commonly a trainer cup and information leaflet) and resource packs (photos of caries, information leaflets, x-rays) were appreciated by HVs and reported to facilitate the conversation about oral health between HVs and parents and enabled the discussion to be more structured [40, 51]. Furthermore, HVs believed resource packs offered an opportunity for interdisciplinary working between themselves and community dental services. Families also appreciated the toothbrushing packs as an oral health resource [51]. As for the resource packs, HVs found photographs and x-rays depicting the development of teeth, dental plaque, and caries particularly useful during their conversations with families [40].

A qualitative study exploring HVs’ and school nurses’ perspectives of promoting oral health in children identified the themes ‘responsibility’, ‘barriers’, and ‘cohesive approach’ [54]. HVs spoke of ‘responsibility’ in terms of their responsibility towards their clients, the responsibility of parents for their children, and the responsibility of policymakers. HVs described the need for a ‘cohesive approach’ to promoting oral health with a more integrated provision of oral health promotion involving all health professionals. Regarding the theme barriers, more detail will be discussed in the following section on barriers encountered by HVs as this was a prevalent theme among several studies.

##### Barriers and facilitators to promoting oral health

Barriers faced by HVs in promoting oral health were explored [32, 51, 52, 54, 55]. Exploring the views of HVs, a lack of effective communication with dental services was identified as a significant barrier HVs faced when supporting families with oral health [52]. It was suggested that referral processes to primary NHS dental services were not as effective as other health services. To overcome this barrier, referral processes to NHS dental services need to be improved to enable HVs to facilitate access to local dental services. Furthermore, this study found many HVs were unaware of specific NHS dental services such as dental services for children with special needs and interpreting services, and thus unable to facilitate dental care access.

Communication barriers were not limited to dental services and were also encountered with families [51]. HVs sometimes faced language barriers and the presence of an interpreter potentially interfered with HVs establishing direct rapport with families. Resources such as visual aids were critical for HVs to facilitate communication.

Moreover, limited oral health and human resources have been cited as barriers. With limited human resources, health visiting services are at times overstretched. Additionally, HVTs reported that not all the required health and developmental topics could be discussed within the allocated duration of the routine check. Consequently, some topics were often prioritised over others depending on the specific needs of each family [55].

Another potential barrier was the confidence of HVs in promoting oral health. The literature suggested that a lack of oral health training impacted negatively on the confidence of HVs in delivering oral health advice [32, 51, 55]. To overcome this barrier they found that HVs, as mentioned previously, needed better oral health knowledge and specifically up-to-date evidence-based recommendations to address gaps in knowledge [32, 51, 55]. In addition to evidence-based oral health information, the literature suggests it is important for HVs to be provided with more culture-specific oral health information and guidance [52].

Conflicting oral health information provided by other health professionals [51] and limited collaborative multi-disciplinary working was a reported barrier to engaging parents in oral health discussions [55].

### Results of the grey literature

#### Characteristics of the included sources

**Fig. 2:**
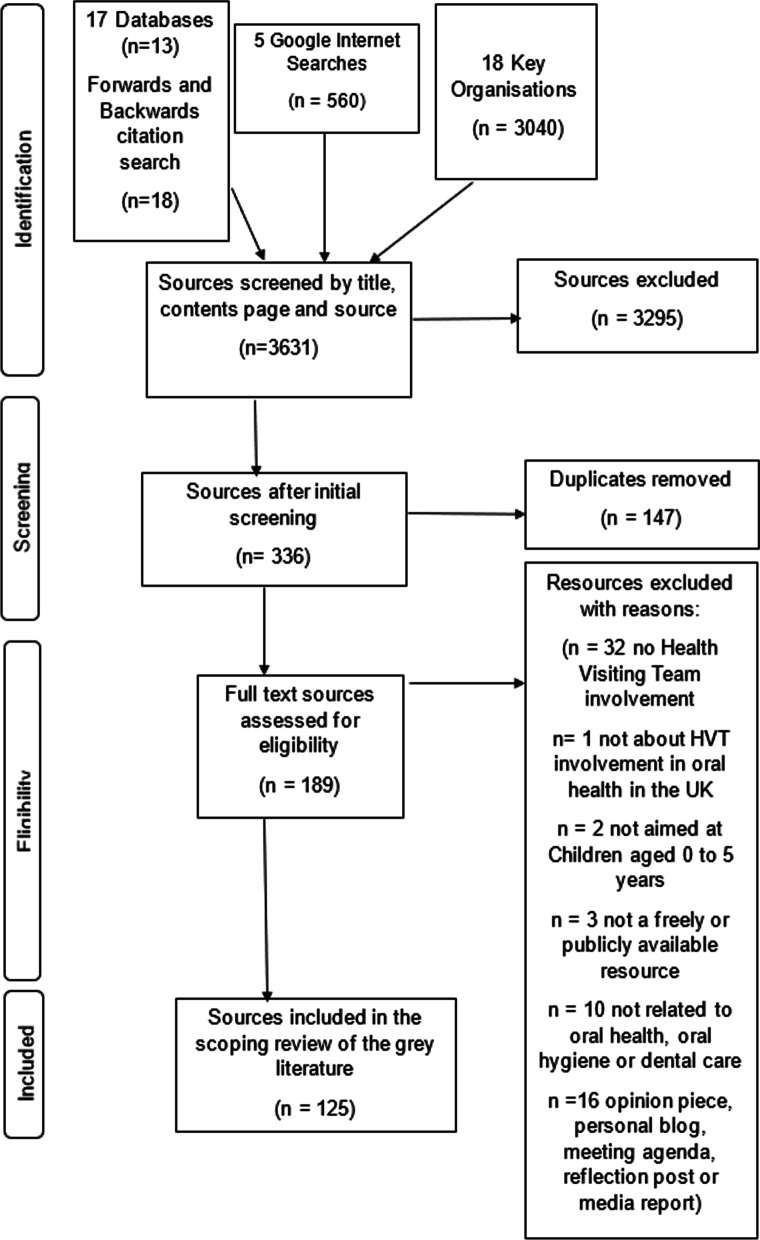
PRISMA-ScR flowchart for the grey literature

Overall, 125 sources of grey literature were included (Additional file 1: Appendix 9). A study flow diagram created using the PRISMA_ScR template and a previous grey literature flow diagram is presented in Fig. 2.

The majority of sources reporting HVTs involvement in oral health were from England (87/125) and Scotland (29/125); Wales (7/125) and Northern Ireland (2/125) had the fewest number of sources included in the review.

A variety of source types reported HVTs involvement in oral health, the most common source types were reports (39/125), guidance documents (29/125), and evaluations (22/125). The least common source types were reviews (4/125) and training resources aimed at HVTs (4/125).

The four countries of the UK differed in source types used to report HVTs involvement in oral health; in England, the most common source types were reports (29/87) and guidance documents (18/87), whereas, in Scotland, the most common source type was evaluation (11/29).

#### Health Visiting Team involvement in Oral Health

The grey literature reported HVT involvement in national and regional oral health improvement programmes: Childsmile, Designed to Smile, Brushing for Life, Starting Well Core, First Dental Steps, and Smile4Life, and suggests HVT involvement in oral health varied across the devolved nations.

**Fig. 3:**
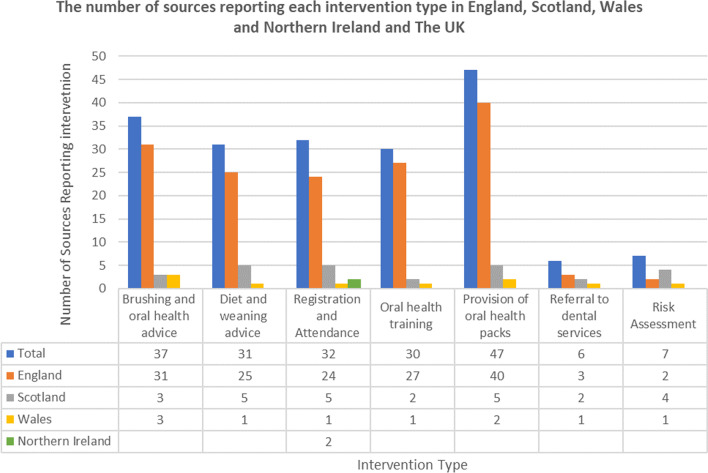
The four nations of the UK and the total number of sources reporting each intervention type

Figure 3 shows the total number of sources reporting each intervention type, the most common intervention, with HVTs involvement, was the provision of oral health packs (n = 47). The least common reported interventions were risk assessment (n = 7) and referral to dental services (n = 6).

HVTs role in promoting registration and attendance to dental services included signposting to local dental practices, encouraging dental attendance before a child’s first birthday, and providing detail on how to access emergency services. Specific examples reported in the literature included the ‘Now you have teeth’ project which was a collaborative project between HVTs and dentists to encourage dental attendance of children before their first birthday [62]. HVTs have also promoted registration and attendance by providing families with a voucher or a dental registration pack; information included how to register children with a dental practice and the importance of maintaining registration [13, 63, 64]. Health visiting centres also displayed posters of the ‘Dental Check by 1’ campaign to promote dental attendance before a child’s first birthday [65].

HVTs provided brushing and oral health advice, they recommended brushing teeth with fluoride toothpaste, ensuring brushing is supervised, brushing as soon as the first teeth erupt, and optimal timing of tooth brushing. A minority of sources (3/125) reported the amount of fluoride to be used at different developmental stages and appropriate fluoride concentrations [13, 66, 67] Brushing and oral health advice by HVTs was a component of Designed to Smile (D2S), and several of England’s OHIPs (Additional file 1: Appendix 10).

Weaning advice provided by HVTs comprised of promotion of breastfeeding, healthy weaning, and using a free-flowing cup by 12 months. Diet advice focused on the promotion of healthy food and snacks. D2S included HVTs discussing the use of sugar-free medicine with families [68]. Case studies in Manchester and Huddersfield highlighted the role of HVs in changing infant feeding practices [69, 70]. In Manchester, HVs were involved in changes to the Healthy Baby Infant Feeding Policy and in Huddersfield, HVs participated in a ‘Beakers for bottle’ event; both initiatives aimed to address culturally embedded prolonged bottle feeding customs [69, 70]. The impact of HVTs providing families with diet diaries on diet and weaning practices was also explored in a study conducted in Scotland [71].

The most commonly reported intervention with HVTs involvement was the provision of oral health packs; packs consisted of fluoride toothpaste, toothbrushes, and leaflets with advice on toothbrushing. A free-flow beaker was also included in some oral health packs distributed by HVTs [13, 70, 72]. The majority of national and regional programmes included the provision of oral health packs (Additional file 1: Appendix 10). Targeted provision of oral health packs by HVTs was suggested to help reduce oral health inequalities in 0-to-5-year-olds [63, 66, 73–75].

Four training resources were identified that were specifically aimed at HVTs these consisted of two e-learning resources that provided information on topics such as risk factors of dental decay, dental registration, attendance, brushing advice, diet, maternal oral health, referral to dental services, and implications of poor oral health for safeguarding [76, 77]. The remaining two resources were an infographic displaying key oral health messages and good practice points for HVTs to consider when providing oral health advice [78, 79].

HVTs’ role in risk assessment and referral to dental services was less widely reported in the sources. D2S, Childsmile, and the Starting Well programme included HVTs facilitating referral to dental services (Additional file 1: Appendix 10). The role of HVTs in risk assessment included HVTs identifying families who may require additional oral health support, for example identifying children with a sibling who has attended hospital for dental extractions due to tooth decay [80]. In Wales, HVs were encouraged to look at children’s teeth as part of the ‘Lift the Lip’ campaign [81]. As part of the campaign, HVs were trained to visually examine the front upper teeth and identify early signs of tooth decay [81]. In Dundee, HVTs, in partnership with dental professionals, were involved with developing a caries risk assessment tool for preschool children [82, 83].

#### Evaluation of oral health interventions which involve HVTs

Qualitative and quantitative outcome measures were used to evaluate HVT involvement in oral health. Quantitative outcome measures were related to the proportion of HVT visits that included oral health, recruitment rates, and the percentage of HVTs who had received oral health training. The outcome measures employed regarding children’s oral health related to obvious dental decay levels, registration with a dentist, access, and the number of decayed, missing or filled (dmft) primary teeth. Intervention cost was reported in two sources [84, 85]. The targeted provision of toothbrushes and toothpaste by HVs was the only oral health intervention with HVTs involvement where cost-effectiveness was reported [80].

## Discussion

As far as the authors are aware this is the first review to map and synthesise the evidence on the role of HVTs in improving children’s oral health. This review aimed to broadly scope the literature available on the contribution of HVTs in improving the oral health of children aged 0–5 years and oral health interventions for children aged 0–5 years that involve HVTs, provide a synthesis of key findings, and recommendations for further research.

There was agreement across all peer-reviewed and grey literature that there is a role for HVs in the promotion of oral health of young children and that the majority of HVs view promoting oral health as important and acceptable. Their role involved delivering oral health advice, toothbrushing packs, and other oral health resources, as well as promoting dental registration and access to dental services to families of young children. Additionally, their role involved supporting nurseries to review policies and practices for a conducive environment for promoting oral health. The success of Childsmile in Scotland emphasises that HVTs have a pivotal role in the oral health improvement of children, especially where integrated referral pathways are in place. Furthermore, the ability to refer families to a professional such as the Dental Health Support Worker in Childsmile could be a timely and efficient method to ensure families receive oral health messages and reduce the burden for HVTs to provide in-depth oral health advice.

The peer-reviewed literature indicated that overall HVs had good knowledge of oral health and provided dental advice during developmental checks. Gaps in knowledge of HVs however were identified particularly concerning fluoride guidelines, toothbrushing guidelines, and toothbrushing behaviour management in very young children. Moreover, only four training resources aimed at HVTs were identified from the grey literature highlighting the scarcity of training resources in oral health aimed at HVTs. The findings of this review suggest there is a need for improved formal education, training, and training resources for HVTs in oral health. The desired outcome is increased oral health knowledge and skills in delivering oral health promotion however it is imperative to appreciate that increased knowledge and skills of HVTs are necessary but not sufficient for changes in practice [86] as there are many contextual factors involved.

Indeed, the findings indicate that HVs encounter several barriers to promoting oral health in children. Significant barriers were reported such as limited resources in terms of both human and oral health resources. HVs have demanding workloads with several tasks to undertake within the specified time of the routine developmental check. These time constraints result in competing priorities with the topic of oral health competing with other health and developmental topics required to be discussed with families. Moreover, the lack of availability of oral health resources for HVs to access renders it more challenging to address oral health issues. Other barriers reported included a lack of confidence in HVs in delivering oral health advice due to a lack of comprehensive oral health education and training; communication barriers with dental services and with parents; and the provision of conflicting oral health advice by other health professionals. Therefore, to enable HVTs to effectively promote oral health a more detailed understanding of key barriers and enablers at various levels such as individual, organisational and policy levels is needed and addressed accordingly.

### Strengths and limitations

A strength of this scoping review is the extensive search of both the peer-reviewed and grey literature; this in-depth focus of the grey literature enabled the inclusion of a range of literature sources providing valuable contextual information on the role of HVTs in oral health improvement. In addition to a comprehensive and reproducible search strategy informed by experts in their field and academics. A limitation of this review relates to terminology. This review included the term health visiting teams acknowledging the practice of health visiting as a team approach. The majority of the included studies of the peer-reviewed search mainly used the term health visitor and occasionally specified whether it was a community nurse or school nurse. The term health visiting teams was found to be used in the more recent publications. As many of the included studies were old this may be a reason for the predominant use of the term health visitor rather than the term health visiting teams. Moreover, although the search strategy for the peer-reviewed literature was not limited to the UK only two of the included studies were from countries other than the UK. This indeed is indicative of the variations in terminology across the globe for professionals carrying out similar roles of HVTs in the UK. Furthermore, due to many of the included studies being old, they do not reflect more recent changes in professional practice and service specification.

Another limitation of this scoping review pertains to the grey literature, in which only a selection of the searches was duplicated due to time constraints. Additionally, data charting was completed by one researcher. This may have resulted in the potential introduction of bias and inaccuracy to the charting process. Likewise, the searches were limited to the UK due to concerns regarding the number of results an international search would yield and differences in HVTs professional roles and service provision in the UK compared with internationally. However, based on the results of the peer-reviewed search which was not limited to the UK it was not anticipated that conducting an international search would have resulted in additional significantly relevant sources. The search strategy produced only two eligible sources from Northern Ireland, concerns regarding this were discussed with two senior research associates and the searches were deemed comprehensive. However, as experts and academics consulted did not include personnel from Northern Ireland key organisations or search terms may have been missed resulting in fewer eligible sources from Northern Ireland.

### Recommendations for future research

Most of the studies included in this review were cross-sectional and used self-reported surveys to evaluate HVs’ knowledge and practice. While this data is useful it may not be an accurate representation of actual practice. Studies that include observation of the actual practice of HVs would strengthen the literature available, in addition to more good quality intervention studies and evaluation studies. Additionally, future studies need to be of strong quality and take account of the inadequacies found in the current research. Future research should also explore the cost-effectiveness of OHIPs involving HVTs and the effectiveness and cost-effectiveness of training HVTs in oral health promotion should be evaluated further. Regarding the sources available across the four devolved nations, Northern Ireland had the fewest sources with a lack of a national or regional OHIP with HVTs involvement. Consequently, further research regarding the role of HVTs in improving the oral health of 0 to 5-year-olds in Northern Ireland should be considered.

## Conclusion

This scoping review has demonstrated the nature and importance of HVTs role in children’s oral health. There appears to be a lack of reviews that have synthesised HVTs involvement in children’s oral health. This scoping review addresses this gap. The findings of this review demonstrate that HVTs were often involved in OHIPs and have general good knowledge of oral health however gaps in education and training exist. Addressing these gaps and other barriers encountered by HVTs is paramount for better enabling HVTs in supporting families in improving their child’s oral health. This scoping review provides a useful starting point to guide future research and areas for systematic reviews.

## Supplementary Information

**Additional file 1**. Appendices 1, 7, 8, 9, 10.

**Additional file 2**. Appendix 2.

**Additional file 3**. Appendices 3, 4, 5, 6.

## Data Availability

The datasets synthesised in the current study are available from the corresponding author upon reasonable request.

## Abbreviations

ECC: Early childhood caries
HVs: Health visitors
HVTs: Health visiting teams
HCP: Healthy Child Programme
OHIP: Oral Health Improvement Programme
RCT: Randomised Controlled Trial
