# Supplementary material for: Health visiting teams and children’s oral health: a scoping review
Source: BMC Oral Health. 2022 Dec 10;22:594. doi: 10.1186/s12903-022-02611-6 (PMC9741786; doi:10.1186/s12903-022-02611-6)
Supplement: Supplementary file 1 — Additional file 1. Appendices 1, 7, 8, 9, 10. [file 12903_2022_2611_MOESM1_ESM.docx]

| **Country** | **The national children’s health programme** | **Target age group** | **HVT role in the programme** | **Inclusion of Oral Health Promotion** |
| --- | --- | --- | --- | --- |
| England | Healthy Child Programme^12^ | 0-to-19 years | 0-to-5 years element delivered by HVTs | The percentage of 5-year old’s with visually obvious decay is one of the key indicators used in the Public Health Outcomes Framework (PHOF) |
| Scotland | Health for All Children (Hall 4)^9^ | 0-to-19 years | HVTs deliver the Universal Health Visiting Pathway aimed at children aged 0-to-5 | HVTs ensure every newborn in Scotland is linked to Childsmile, the national oral health improvement programme, and provided oral health promotion |
| Wales | Healthy Child Wales Programme (HCWP)^11^ | 0-to-7 years | The HCWP is delivered by HVTs and school nursing teams | The percentage of 5-year old’s with visually obvious decay is one of the key indicators used in the PHOF |
| Northern Ireland | Healthy Child, Health Future (HCHF)^10^ | 0-to-19 years | Health professionals including midwives, HVs and general practitioners deliver the Universal Preschool Programme from 12 weeks of pregnancy to 4 ½ years | Promotion of oral health is one of the key topics to be covered in the HCHF and is a suggested topic of health promotion at several mandated visits with HVTs |

**Appendix 1.** A summary of the national children’s health programme in each of the four nations of the UK

**Appendix 7**. Table of included studies – peer-reviewed literature

| First author, year/country | Title | Method of data collection | Aim |
| --- | --- | --- | --- |
| Kipping, 2016 /UK | NAP SACC UK: protocol for a feasibility cluster randomised controlled trial in nurseries and at home to increase physical activity and healthy eating in children aged 2–4 year | Feasibility cluster RCT (Protocol) | Examine the feasibility and acceptability of adapting the Nutrition and Physical Activity Self-Assessment for Child Care (NAP SACC) intervention in the UK to inform a full-scale trial. |
| Langord, 2019 /UK | A physical activity, nutrition and oral health intervention in nursery settings: process evaluation of the NAP SACC UK feasibility cluster RCT | Process evaluation embedded in RCT | Assess feasibility & acceptability to key stakeholders. |
| Arrow, 2013 /Australia | Brief oral health promotion intervention among parents of young children to reduce early childhood dental decay | RCT (Protocol) | Assess if an early oral health promotion intervention using motivational interviewing & anticipatory guidance approaches can reduce the incidence of early childhood dental decay and obesity. |
| Hamilton,1999/UK | An oral health promotion programme for nursing caries | Cross-sectional | To evaluate an oral health promotion programme involving health visitors and mothers of 8-month‐old babies to address some of the risk factors associated with nursing caries. |
| Eskyte,2018 /UK | HABIT—an early phase study to explore an oral health intervention delivered by health visitors to parents with young children aged 9–12 months: study protocol | RCT (Protocol) | To explore the acceptability of the HABIT intervention to parents and health visitors, to examine the mechanism of action and develop suitable objective measures of parental supervised brushing |
| Chadwick, 2005 /UK | Primary care research: difficulties recruiting preschool children to clinical trials. | RCT | Report difficulties experienced recruiting preschool children to a clinical trial. Report the acceptability of a dental intervention to their parents |
| Turner, 2010 /UK | Childsmile: the national child oral health improvement programme in Scotland. Part 2: monitoring and delivery | Longitudinal | To monitor the delivery of the Childsmile programme |
| Williams S, 1982/ UK | Health visitors and dental health education | Cross-sectional | To discover how much previous dental health teaching had been provided for a group of practising health visitors. |
| Oge,2018/UK | Knowledge, attitude and practice among Health Visitors in the United Kingdom toward children's oral health. | Cross-sectional | To determine knowledge, attitude, and practical behaviour of health visitors regarding children’s oral health in the UK |
| Doughty,2016/ UK | Challenges identified in a pilot outreach dental service for Traveller children in Hackney, East London. | Feasibility study | To work collaboratively with the community nurse responsible for the Traveller community based within Hackney council: to ensure that the Traveller children had access to dental prevention and oral care; to assess the extent of any oral problems, to engage with the Traveller families on site and to evaluate the outcomes of a pilot outreach dental service |
| Hunter,1998/UK | Oral health advice: reported experience of mothers of children aged 5 years and under referred for extraction of teeth under general anaesthesia. | Cross-sectional | To examine the reported receipt of oral health education among mothers of children who required the extraction of teeth under general anaesthesia. |
| Lewney,2019/UK | Health visitors' views on promoting oral health and supporting clients with dental health problems: a qualitative study. | Qualitative | To explore how health visitors felt about providing oral health advice and dealing with dental issues during their practice. |
| Whittle, 2008/UK | A randomised control trial of oral health education provided by a health visitor to parents of pre-school children. | RCT | To determine the effect of oral health education carried out by a specially trained health visitor on the dental health of young children |
| Williams,N.  2002 /UK | The relationship between socio-demographic characteristics and dental health knowledge and attitudes of parents with young children. | Cross-sectional | To determine if parental socio-demographic characteristics are associated with dental knowledge and attitude. |
| Yuan, 2007/UK | Evaluating a community-based dental registration program for preschool children living in areas of high social deprivation. | Quasi-experimental comparison | To evaluate the effectiveness of a community-based program to promote dental registration and access to dental services for preschool children residing in areas of high social deprivation. |
| Bentley,1994/UK | Dental health. I. Views about preventive dental care for infants. | Cross-sectional | To investigate attitudes of HVs and parents to preventive dental health care for young children. |
| Ingram,1987/UK | Dental health factsheets for health visitors and other health professionals. | Qualitative | To develop dental factsheets for non-dental health practitioners (Camberwell scheme). |
| Quinn, 1994/UK | Dental health. 2. Working together in dental health education | Cross-sectional | Examine the interactions between HVs and community dental officers to discover of cooperation influences provision of dental health education |
| Bentley, 1993/ UK | An evaluation of the role of health visitors in encouraging dental attendance of infants | Longitudinal | To measure the number of children aged 0-2 year registered as a result of the campaign. To evaluate the effectiveness of health visitors in increasing dental attendance among this age group and to collect the views of the dentists and health visitors on the conduct and outcome of the campaign. |
| Williams, 1984/ UK | Health visitors and dental health awareness | Cross-sectional | To determine the level of awareness about dental health among practising health visitors. |
| Hunter, 1996/ UK | The current status of dental health education in the training of midwives and health visitors | Cross-sectional | Investigate the current status of dental health in the education of mid-wives &health visitors. |
| Davies, 1993/UK | An investigation into the role of post-natal health clinics in oral health education | Cross-sectional | 1. Assess if parents in one UK health district were receiving OH education in post-natal clinics and what information had been given, by who and the impact on parents' knowledge and awareness of OH. |
| Bentley,1997/ UK | The rationale, organisation and evaluation of a campaign to increase the use of sugar-free paediatric medicines. | Longitudinal | To conduct & evaluate a dental health education campaign to encourage the use of sugar free medicine. |
| McKeown,2003/UK | Evaluation of an oral health resource pack for health visitors | Cross-sectional | To evaluate a resource pack developed for HVs to use with the parents of young children. |
| Weston-Price,2020/UK | Barriers and facilitators to health visiting teams delivering oral health promotion to families of young children: a mixed methods study with vignettes. | Mixed-method | To explore the potential barriers and facilitators to health visiting teams delivering oral health promotion during the 9-12-month old child mandated visit in Ealing, England |
| Kipping,2019/UK | Child-care self-assessment to improve physical activity, oral health and nutrition for 2-to 4-year-olds: a feasibility cluster RCT | RCT | To evaluate the feasibility and acceptability of implementing NAP SACC in the UK. |
| Glatt,2015/USA | Evaluation of an oral health education session for EarlyHead Start home visitors | Before - After comparison | To conduct and evaluate an educational intervention that improved oral health knowledge and communication techniques among Wisconsin EHS home visitors. |
| Coll,2016/UK | Health visitors’ and school nurses’  perceptions of promoting dental  health in children | Qualitative | To explore the views of health visitors and school nurses regarding their role in oral health promotion and their understanding of the issues surrounding the delivery of effective oral health promotion in their daily practice. |
| Quinn, 1991/UK | Health visitors as dental health educators: their knowledge, attitudes and behaviours | Cross-sectional | To investigate the levels of dental knowledge, attitudes and personal health and working behaviours in a group of health visitors, to assess their ability to conduct dental health education. |
| Macpherson, 2010/UK | Childsmile: the national child oral health improvement programme in Scotland. Part 1: Establishment and development. | Longitudinal | To describe the development and implementation of this national oral health improvement programme for children in Scotland over its initial three-year period (January 2006 to December 2008) and how monitoring and evaluation are shaping the delivery and direction of the programme. |
| Macpherson, 2019/UK | Childsmile after 10 years part 2: programme development, implementation and evaluation. | Longitudinal | To improve the oral health of children in Scotland and reduce inequalities both in dental health and access to dental services. |
| Williams,1980/UK | Dental health teaching and health visitors. | Cross-sectional | To discover whether and to what extent dental health was included as a separate topic in the curricula of the training colleges of England and Wales. |
| Davies G.,2005/UK | A staged intervention dental health promotion programme to reduce early childhood caries | RCT | to assess the effects of a mult-stage dental health promotion programme in reducing ECC |
| Davies, K.,1992/UK | An assessment of dental health education in a baby clinic | Cross-sectional | Recall of advice being given and change on oral health behaviours and dmft |
| Wlliams S,1984/UK | Attitudes of health visitors to fluoridation | 2 Cross-sectional surveys | to determine the attitudes of a large sample of health visitors working in urban communities in the North and the South of England at two distinct points in time |
| Griffiths C,1985/UK | The heath visitor and dental health education | Cross-sectional | Use a questionnaire delivered to HVs and expectant mothers to design an appropriate talk to improve their dental awareness |
| Anopa Y,2015/UK | Improving Child Oral Health: Cost Analysis of a National Nursery Toothbrushing Programme | Economic evaluation | To compare the cost of providing the Scotland-wide nursery toothbrushing programme with associated National Health Service (NHS) cost savings from improvements in the dental health of five-year-old children: through avoided dental extractions, fillings and potential treatments for decay. |
| Wilson, M,2019/UK | Introducing 'Lift the Lip'into the routine practice of health visitors | Feasibility study | To assess the feasibility and acceptability of introducing Lift the Lip into the routine practice of health visitors in Wales. |
| Eskyte I, | Organizational Barriers to Oral Health Conversations Between Health Visitors and Parents of Children Aged 9-12 Months Old. | Qualitative | Our study explored the organizational factors that obstruct health visitors from engaging in meaningful conversations with parents about young children's oral health. |

**Appendix 8**. Table of studies included in quality assessment

| First author, year/country | Title | Overall global rating |
| --- | --- | --- |
| Chadwick, 2005 /UK | Primary care research: difficulties recruiting preschool children to clinical trials. | Weak |
| Bentley, 1993/ UK | An evaluation of the role of health visitors in encouraging dental attendance of infants | Moderate |
| Whittle, 2008/UK | A randomised control trial of oral health education provided by a health visitor to parents of pre-school children. | Moderate |
| Yuan, 2007/UK | Evaluating a community-based dental registration program for preschool children living in areas of high social deprivation. | Strong |
| Bentley,1997/ UK | The rationale, organisation and evaluation of a campaign to increase the use of sugar-free paediatric medicines. | Moderate |
| Kipping,2019/UK | Child-care self-assessment to improve physical activity, oral health and nutrition for 2-to 4-year-olds: a feasibility cluster RCT | Weak |
| Davies G.,2005/UK | A staged intervention dental health promotion programme to reduce early childhood caries | Strong |

**Appendix 9.** Table of included sources – grey literature

| **Author and Year** | **Document Type** | **Country of Publication** | **Name of Programme** |
| --- | --- | --- | --- |
| An appraisal of Brushing for Life | Evaluation | England | Brushing for Life (Brushing4Life) |
| Bazian 2013 | Evaluation | Scotland | Childsmile |
| Bazian 2014 | Guidance | England | NS |
| Beverley Green 2017 | Guidance | Scotland | Childsmile |
| Bolton Council | Report | England | Brushing4Life |
| Calderdale Council and Kirklees Council 2019 | Policy | England | NS |
| Cardiff University 2015 | Evaluation | Wales | Designed to Smile (D2S) |
| Cardiff University 2018 | Evaluation | Wales | D2S |
| Childsmile 2016 | Guidance | Scotland | Childsmile |
| Childsmile | Guidance | Scotland | Childsmile |
| City of Bradford Metropolitan District Council 2014 | Guidance | England | NS |
| Darlington Borough Council 2017 | Report | England | NS |
| Dental Health Need Assessment Wirral Summary | Report | England | NS |
| Department of Health 2009 | Report | England | Healthy Child Programme (HCP) |
| Department of Health 2009 | Policy | England | HCP |
| Designed to Smile 2021 | Guidance | Wales | D2S |
| Durham City Council 2017 | Report | England | NS |
| e-Learning for Healthcare | Training | England | NS |
| Faith Hodgkins 2017 | Evaluation | Scotland | Childsmile |
| **Author and Year** | **Document Type** | **Country of Publication** | **Name of Programme** |
| Families and Children's Trust Northumberland 2010 | Guidance | England | NS |
| Further Update On The Progress Of The Oral Health Strategy And The Dental Action Plan 2009 | Evaluation | Scotland | Childsmile |
| Health and Wellbeing Scrutiny Committee 2018 | Report | England | NS |
| Health Education England 2016 | Training | England | NS |
| Healthwatch Brent 2018 | Evaluation | England | NS |
| Heather Ballantyne-MacRitchie 2000 | Evaluation | Scotland | NS |
| Hi-Net Grampians Health Improvement Network | Report | Scotland | Childsmile |
| Hull City Council 2015 | Policy | England | Brushing4Life |
| Information Analysis Directorate 2019 | Report | Northern Ireland | Healthy Child, Healthy Future |
| Institute of Health Visiting 2015 | Training | England | NS |
| Integrated Public Health Nursing Service 2017 | Review | England | NS |
| Jamie Brian Rutherford Kidd 2019 | Evaluation | Scotland | Childsmile |
| Knowsley Council 2015 | Report | England | NS |
| Leeds City Council 2015 | Report | England | Brushing4Life |
| Liverpool Public Health Observatory 2015 | Report | England | NS |
| Local Government Association 2016 | Case Study | England | NS |
| Local Government Association 2016 | Case Study | England | Building Brighter Smiles and Suffolk Smiles |
| Local Government Association 2016 | Case Study | England | Building Brighter Smiles |
| Local Government Association 2016 | Case Study | England | Suffolk Smiles |
| Local Government Association 2017 | Case Study | England | Hull City Council: a focus on improving oral health |
| Local Government Association 2018 | Case Study | England | Starting Well |
| **Author and Year** | **Document Type** | **Country of Publication** | **Name of Programme** |
| Local Government Association 2019 | Case Study | England | Hull City Council: a focus on improving oral health |
| London Borough of Barking and Dagenham 2016 | Policy | England | NS |
| London Borough of Hammersmith & Fulham 2013 | Report | England | Brushing4Life |
| London Borough of Hounslow 2013 | Policy | England | Brushing4Life |
| Lorna Macpherson | Evaluation | Scotland | Childsmile |
| Mairi Anne Young 2017 | Evaluation | Scotland | Childsmile |
| Mary Kimberly Wilson 2012 | Evaluation | Scotland | NS |
| Medway NHS Primary Care Trust 2006 | Report | England | NS |
| National Assembly for Wales Children and Young People Committee 2012 | Report | Wales | D2S |
| National Assembly for Wales Health, Social Care and Sport Committee 2019 | Policy | Wales | D2S |
| National Institute of health and Care Excellence (NICE) 2014 | Guidance | England | NS |
| NHS Bradford and Airedale 2021 | Evaluation | England | Brushing4Life |
| NHS Bury Clinical Commissioning Committee 2019 | Report | England | The 0-5 Greater Manchester Oral Health Transformation programme |
| NHS England 2014 | Policy | England | NS |
| NHS England 2019 | Guidance | England | Starting Well |
| NHS England and Leicester City Council 2014 | Report | England | NS |
| NHS England and NHS Improvement – East of England 2019 | Report | England | NS |
| NHS England and NHS Improvement 2021 | Report | England | NS |
| NHS Greater Glasgow 2005 | Report | Scotland | Oral Health Promotion in Glasgow |
| NHS Greater Glasgow and Clyde 2012 | Evaluation | Scotland | Childsmile |
| **Author and Year** | **Document Type** | **Country of Publication** | **Name of Programme** |
| NHS Greater Glasgow and Clyde 2013 | Evaluation | Scotland | Childsmile |
| NHS Health Scotland 2004 | Report | Scotland | NS |
| NHS Health Scotland 2009 | Evaluation | Scotland | Childsmile |
| NHS Rotherham Doncaster and South Humber | Guidance | England | NS |
| NHS Scotland 2003 | Policy | Scotland | Lothian Pre-School Registration and Scottish Oral Health Demonstration Project |
| NHS Scotland 2012 | Guidance | Scotland | NS |
| NHS Scotland 2018 | Guidance | Scotland | Childsmile |
| NHS Tayside 2010 | Report | Scotland | Childsmile |
| NHS Tayside 2013 | Report | Scotland | Childsmile |
| NHS Tayside 2014 | Report | Scotland | Childsmile |
| NHS Wales 2002 | Policy | Wales | NS |
| NHS Wales 2013 | Report | Wales | D2S |
| NICE 2007 | Evaluation | England | NS |
| NICE 2008 | Guidance | England | NS |
| NICE 2013 | Guidance | England | Brushing4Life and Childsmile |
| NICE 2013 | Guidance | England | NS |
| NICE 2014 | Guidance | England | NS |
| NICE 2014 | Guidance | England | NS |
| NICE 2014 | Guidance | England | NS |
| NICE 2016 | Guidance | England | NS |
| NICE 2016 | Policy | England | NS |
| **Author and Year** | **Document Type** | **Country of Publication** | **Name of Programme** |
| Northern Ireland Personal Child Health Record (PCHR) Professional Guidance On Record Completion 2010 | Guidance | Northern Ireland | NS |
| Officer for Public Health in Scotland 1999 | Report | Scotland | NS |
| Oldham Council 2018 | Report | England | NS |
| Oral Health Improvement Service | Policy | England | NS |
| Oral Health Promotion 2019 | Guidance | England | NS |
| Oral Health Promotion Strategy development group 2016 | Policy | England | Brushing4Life |
| Plymouth City Council 2019 | Report | England | First Dental Steps |
| Public Health England 2014 | Guidance | England | NS |
| Public Health England 2015 | Policy | England | Brushing for Smiles |
| Public Health England 2015 | Review | England | Brushing4Life and Five-Step approach in Manchester |
| Public Health England 2016 | Training | England | NS |
| Public Health England 2016 | Policy | England | Oral Health Improvement Programme |
| Public Health England 2016 | Review | England | Oral Health Improvement Programme |
| Public Health England 2016 | Guidance | England | NS |
| Public Health England 2017 | Report | England | NS |
| Public Health England 2017 | Guidance | England | NS |
| Public Health England 2017 | Case Study | England | Smile4Life |
| Public Health England 2017 | Case Study | England | Happy Teeth, Happy Smiles! |
| Public Health England 2018 | Evaluation | England | NS |
| Public Health England 2019 | Guidance | England | NS |
| Public Health England 2021 | Report | England | NS |
| **Author and Year** | **Document Type** | **Country of Publication** | **Name of Programme** |
| Public Health England 2021 | Guidance | England | Healthy Child Programme (HCP) |
| Public Health Kingston 2018 | Report | England | NS |
| Rochdale Borough Council 2018 | Report | England | Brushing4Life |
| Royal College of Nursing 2019 | Report | England | NS |
| Salford City Council 2019 | Evaluation | England | NS |
| Scottish Dental Clinical Effectiveness Programme 2018 | Guidance | Scotland | Childsmile |
| Scottish Government 2015 | Guidance | Scotland | Universal Health Visiting Pathway in Scotland |
| Scottish Intercollegiate Guidelines Network 2014 | Guidance | Scotland | Childsmile |
| Somerset County Council 2015 | Evaluation | England | NS |
| South Tyneside Council 2019 | Policy | England | NS |
| Southwark Council 2018 | Report | England | NS |
| Surrey-I 2021 | Report | England | NS |
| The Oral Health Advisory Group (OHAG) in Hull 2015 | Policy | England | Brushing4Life |
| The Oral Health Advisory Group (OHAG) in Hull 2016 | Review | England | Brushing4Life |
| Tizzy Keller 2018 | Report | England | NS |
| Tower Hamlets NHS 2015 | Report | England | Brushing4Life |
| UK Research and Innovation 2017 | Evaluation | England | NS |
| University College London (UCL) 2018 | Evaluation | England | Brushing4Life |
| Wakefield Council 2017 | Report | England | Brushing4Life |
| Welsh Government 2017 | Policy | Wales | D2S |
| Word of Mouth 2014 | Report | England | NS |
| **Author and Year** | **Document Type** | **Country of Publication** | **Name of Programme** |
| Year 3 2019/20 Progress Report – Master 2019 | Report | Scotland | Childsmile |
| York Health Economics Consortium 2014 | Guidance | England | NS^[[1]](#endnote-1)^ |

**Appendix 10.** A summary of the national and regional OHIP with HVTs involvement in Scotland, Wales, and England

1. | **Country** | **Name of Programme** | **National or Regional** | **Nature of Intervention(s)** | **Details of HVTs involvement** | **Launch Year and Duration** | **Targeted or Universal** |
   | --- | --- | --- | --- | --- | --- | --- |
   | Scotland | Childsmile | National | - Promotion of attendance to and registration with a dental practice - Risk assessment - Provision of oral health packs - Referral to dental services | - HV refers child to a Childsmile Dental Health Support Worker (DHSW) at the 6-8 week assessment - DHSW then support families with registering and attending dental services. - 27- 30 month review HVs discuss child oral health status, dental registration, and attendance in the last 12 months - Childsmile referral status checked through a pre-populated review form. - By 5-years, every child is provided with a dental pack containing a toothbrush, a tube of 1000ppm fluoride toothpaste, and an information leaflet on up to six separate occasions. - These occasions are as follows: every child between 8 months to 1 year, all infants aged 1-3 years in areas of deprivation, children at nursery aged 3-4 years and children at the start of primary school | Interim demonstration phase: 2008  Fully integrated Childsmile model: 2011  Duration: 13 years | Universal with additional oral health packs targeted at infants in areas of deprivation |
   | Wales | Designed to Smile (D2S) | National | - Diet and weaning advice - Brushing and oral health advice - Promotion of attendance to and registration with a dental practice - Referral to dental services - Risk assessment - Provision of oral health packs - Oral health training | - As part of the D2S programme HVTs promote breastfeeding, healthy weaning and a healthy diet, and provide brushing and oral health advice. - HVTs also provide eligible families with information on how to access routine and emergency services and can refer children in pain or with signs of infection directly to the D2S team for care. - Practical demonstration by HVs to parents and carers of how to look after children’s teeth - Every eligible child is provided with an oral health pack at 6 months by HVTs with additional oral health packs provided at home visits to children in deprived areas | Pilot: 2008 (North and South Wales)  Launched nationally: 2009  Duration: 12 years | Targeted at children in Wales with the highest oral health need. |
   | **Country** | **Name of Programme** | **National or Regional** | **Nature of Intervention(s)** | **Details of HVTs Involvement** | **Duration** | **Targeted or Universal** |
   | England | Brushing for Life | Regional (LA’s with highest levels of tooth decay) | - Provision of oral health packs - Promotion of attendance to and registration with a dental practice - Oral health training - Diet and weaning advice - Brushing and oral health advice | - HV led programme that promotes regular brushing of children’s teeth using fluoridated toothpaste. - The programme involves providing dental packs containing a toothpaste of 1000ppm fluoride distributed to the parents/carers of all infants at their 3-4 month, 9-12 and 2–2.5 years development checks - Advises a specific contact point to provide diet and weaning advice when an infant is 6-9 months old - The provision of timely and evidence based preventative oral health advice by HVs, signposting to local NHS dental services and oral health training to front line healthcare staff. | Launched in: 2001  Duration: NS | Targeted at disadvantaged areas of England. |
   | England | Starting Well Core (0 to 2 years) | National | - Referral to dental services - Promotion of attendance to and registration with a dental practice - Brushing and oral health advice | - HVs support signposting and referrals to practices participating in Starting Well Core and accepting new child patients. - Health Visitors encourage and assist parents of 0-3 year olds to take their child to the dentist as soon as their teeth appear by helping book appointments if needed. - Possible opportunities for HVs to refer children or contact participating practices directly to support families/carers book appointments (e.g. via secure NHS.net email accounts). | Launched: April 2018  Duration: 3 years | Universal |
   | England | First Dental Steps | Regional (South West of England) | - Oral health training - Diet and weaning advice - Brushing and oral health advice - Promotion of attendance to and registration with a dental practice - Referral to dental services - Provision of oral health packs | - HVs will be trained to provide families with evidence-based advice on attendance to the dentist, diet and weaning regimes, oral hygiene practices and signposting to local dental services. - HV’s will also distribute tooth brushing packs to families who receive Universal Plus or Universal Partnership Plus tiers of support. - A direct care pathway will be set up, to allow HVs to refer families at high risk of developing tooth decay to community dental services, where they can access specialist preventative advice and treatment. | Launched: January 2020  Duration: 18 months | Universal with oral health packs targeted at vulnerable families receiving higher tier of support from HVTs |
   | England | The 0-5 Greater Manchester Oral Health Transformation programme | Regional (Greater Manchester) | - Provision of oral health packs - Brushing and oral health advice - Diet and weaning advice | - HVs are trained in oral health and provide oral health advice - HVs offer a dental care pack at their mandated health visits, supporting parents to introduce healthy eating, toothbrushing and encourages dental attendance. | NS | Targeted to the four localities with the highest oral health needs |
   | **Country** | **Name of Programme** | **National or Regional** | **Nature of Intervention(s)** | **Details of HVTs involvement** | **Duration** | **Targeted or Universal** |
   | England | Smile4Life | Regional (North West of England) | - Provision of oral health packs - Oral health training | - Smile4Life toothpaste and brush packs are distributed - Local HVs all receive Smile4Life training to ensure only evidence informed advice is given. | NS | NS |
   | England | Happy Teeth, Happy Smiles! | Regional (Leicester) | - Provision of oral health packs - Diet and weaning advice - Promotion of attendance to and registration with a dental practice - Brushing and oral health advice | - Modelled on Scotland’s Childsmile programme - Free-flow cups, toothbrush and toothpaste packs were distributed, with appropriate advice and information on oral hygiene, diet and early dental attendance by all HVs. - The local Personal Child Health Record (Red Book) has been amended to include Healthy Teeth, Happy Smiles! oral health pages. - Oral health pathways agreed with health visitors and school nurses | Launched in: 2014  Duration: 3 years | Universal with targeted elements |
   | England | Building Brighter Smiles | Regional (Bradford) | - Diet and weaning advice - Brushing and oral health advice - Oral health training - Provision of oral health packs | - Partnership working with HVs and providing breast feeding advice - Training and regular updates in evidence based oral health practice to professionals working with children and young people - HV’s distribute fluoride toothpaste and toothbrushes and provide evidence based oral health advice | NS | Universal and targeted elements |
   | England | Suffolk Smiles | Regional (Suffolk) | - Provision of oral health packs - Diet and weaning advice - Brushing and oral health advice - Promotion of attendance to and registration with a dental practice | - Oral health pack to be given at every 8-9 month check. The pack will include a child’s toothbrush, fluoride toothpaste and a leaflet about oral health and reducing sugar intake. - HVs also give advice on diet, feeding and weaning, caring for children’s teeth, tooth brushing and how to find a local dentist. | Launched: 2015  Duration: 5 years | Universal |

   [↑](#endnote-ref-1)
