# Supplementary material for: Health visiting teams and children’s oral health: a scoping review
Source: BMC Oral Health. 2022 Dec 10;22:594. doi: 10.1186/s12903-022-02611-6 (PMC9741786; doi:10.1186/s12903-022-02611-6)
Supplement: Supplementary file 3 — Additional file 3. Appendices 3, 4, 5, 6. [file 12903_2022_2611_MOESM3_ESM.docx]

**Appendix 3.** Search strategy for peer-reviewed literature

Database: Ovid MEDLINE(R) and Epub Ahead of Print, In-Process, In-Data-Review & Other Non-Indexed Citations and Daily <1946 to June 10, 2021>

1 exp Community Health Nursing/ or exp Nurses, Community Health/ or health visitor*.mp.

2 oral health.mp. or exp Oral Health

3 exp Oral Health/ or exp Dental Care/ or dental health.mp. or exp Oral Hygiene

4 exp Health Knowledge, Attitudes, Practice/ or oral health education.mp. or exp Health Education, Dental

5 oral health promotion.mp. or exp Health Promotion

6 2 or 3 or 4 or 5

7 health visitor*.tw.

8 community health nurse*.tw.

9 health visiting team*.tw.

10 7 or 8 or 9

11 6 and 10

**Appendix 4.** Key organisations suggested by experts in their field and academics

Five experts and academics were identified through the authors to provide suggestions of key organisations and resources they considered be relevant to the grey literature search. The experts were a consultant in Dental Public Health, Oral Health Local Authority lead, an Information Specialist and two Research Associates. After consultation the following key organisations were suggested:

1. Public Health England
2. Association of Directors of Public Health
3. Institute of Health Visiting
4. NHS England
5. British Association for the Study of Community Dentistry
6. National Institute for Health and Care Excellence (NICE)
7. British Association of Paediatric Dentistry
8. Public Health Wales
9. NHS Scotland
10. Public Health Agency (Northern Ireland)
11. Royal College of Paediatrics and Child Health
12. Open Grey
13. British Dental Association
14. Scottish Dental Clinical Effectiveness Programme
15. Scottish Intercollegiate Guidelines Network
16. Local Government Association
17. Royal College of Nursing
18. POST: Bridging research and policy

The search bar for each key organisation website was used and a broad and iterative search of the website was conducted. Various combinations of the following key words and phrases: “health visitor” OR “public health nurse” AND “oral” or “dental” were used. Searches of the key organisations were also verified by completing an additional search on the internet search engine Google in the advanced setting using the following terms and including the site domain to ensure all potentially relevant sources were found: (oral or dental or oral health or dental care), "health visitor" OR "health visiting team" OR "public health nurse" site:

**Appendix 5*.*** Bibliographic and thesis databases searched

1. Ovid Embase
2. CINAHL
3. PsycINFO
4. Social Care Online
5. Proquest Dissertations & Theses Global
6. Electronic Theses Online Service ‐ British Library
7. DART ‐ Europe e‐theses Portal
8. Networked Digital Library of Theses and Dissertations
9. Allcatsrgrey
10. Open Access These and Dissertations
11. Health Evidence
12. Epistemonikos
13. Database of promoting health effectiveness reviews
14. Centre for Reviews and Dissemination Databases
15. National Institute for Health Research Journals Library – Health Technology Assessment
16. Campbell Collaboration
17. Cochrane Library

**Appendix 6.** Google search strategy

The following keywords and phrases were used: (oral or dental or oral health or dental care) AND (health promotion OR knowledge OR health education OR oral health promotion) "health visitor" OR "health visiting team" OR "public health nurse" excluding the term “job”. The five searches differed by inclusion of AND “England”, “Wales”, “Scotland” and “Northern Ireland” for four of the searches and no country specified for the fifth search.

The first ten pages of all Google searches were screened for potentially relevant sources, once the tenth page was reached screening continued and the search stopped when there had been one page of results with no relevant sources.
